# The Relation of Medical Men to Nurses

**Published:** 1912-06-08

**Authors:** 


### THE BRISTOL HEALTH WEEK.

### The Relation of Medical Men to Nurses.

### Dr. D. S. DAVIES, Medical Officer of Health for Bristol, gives his Views.

The Bristol Health Week, which begins immediately after this number of The Hospital goes to press, is of particular interest because of the great combined effort which has been made by the two professions of medicine and nursing to secure its success. This combination, however, though habitually employed in matters of disease and treatment, raises the vital question of the relation of medical men to nurses in its general aspect, and it was to gain his ideas and experience on this point, so important to both professions, that our Commissioner visited Dr. D. S. Davies the other day.

#### Health Weeks in General.

"As you know," Dr. Davies began, "it had been decided almost before the idea of the National Health Week was even heard of that a Conference and Exhibition should be held at Bristol in June this year under the auspices of the Nurses' Social Union. When, however, I was approached as Medical Officer of Health for the city as to the feasibility of holding a National Health Week in Bristol, I ventured to point out that such a project would gain enormously in practical value if it were linked on to a practical organisation of this kind. Critics, however, of this opinion were not wanting; their criticism took the form of arguing that the Health Week in Bristol should synchronise with the Health Weeks that have been held earlier in the year in other towns. I thereupon wrote to the *Western Daily Press* on May 2 pointing out that there was no cause for regret in delaying the project till a date when it could be linked on 'to the unique collection of exhibits bearing upon health questions already possessed by the Nurses' Social Union, which forms a solid nucleus round which the lessons of a Health Week may be grouped.' I am in favour of anything which has a definite practical object rather than of depending upon the chance organisation of a Health Week *ab initio*.

"By exploiting the now popular idea of a Health Week, when united with such a practical organisation as the Nurses' Social Union, I am convinced that the idea will gain in force, and the organisation in popularity and fruitfulness."

"The programme reveals striking co-operation between the medical profession and the nurses?"

"Yes; and I am heartily in favour of this co-operation in those subjects which nurses or lady doctors can best explain to the mothers. These may be summed up, broadly speaking, in the feeding and care of children. Miss Townsend, a member of the Bristol Education Committee, who presides over an admirable school for mothers, is going to lecture on 'The Life of the School Child'; and Mrs. Barnes, C.M.B., is dealing with that terrible problem of the public medical officer, 'Infantile Mortality.' The good which we are aiming at by this co-operation is the kind of good which nurses who visit homes can teach mothers. The popularity which the Health Week can give to the methods of preventing such home-invading evils as infantile mortality, it cannot give to such less individually understood evils as the communicable diseases.

"To be valuable, lessons on such a topic as parasitic disease must be technical: the proper feeding and care of infants can be explained at a mothers' meeting. Indeed, it is to turn the collected homes of Bristol into a kind of mothers' meeting that this part of the programme will endeavour quite rightly to do."

"Technical subjects, however, have not been avoided?"

"No; for the technicality of a subject, from the point of view of the public health, depends not so much upon its nature as upon the interest or otherwise of the public mind towards it. Consumption has not ceased to be a very technical and difficult subject, but the public attention is sufficiently attracted to it to enable Dr. J. E. Squire, C.B., to discourse most usefully upon the best methods to employ for its prevention. He will, no doubt, make it quite clear to the Bristol man in the poor home that while consumption, at any rate in all but incipient cases, cannot always be 'cured,' it can certainly be controlled. And what a Medical Officer understands by control, the public will understand as cure, and gain not a little in prudence and hopefulness by this substitution. This control can be, indeed is, actually beginning to be secured by means of dispensary work. This work will undoubtedly be popularised by the Health Week. Dr. Phillip, who is the centre of this effort in Edinburgh, and Dr. Faill, of the Tuberculosis Dispensary in Bristol, will, no doubt, be helped considerably, and the new dispensary which has been started, as The Hospital noted at the time, will get into touch with a still wider circle of people. That is, of course, the first essential point. It is also a good example of how the idea of a Health Week gains in practicality by being organised in connection with such a practical work."

#### The Doctor on the Nurse.

"Then as to the general relation of the doctor to the nurse?"

"From the medical point of view nurses have two uses, each perfectly distinct in kind. They have an institutional function, and they have a public health, or it may be called, perhaps, a general municipal function. Once the great principle is established that nurses must not usurp medical functions, their sphere of usefulness in relation to medical men is clear enough. In the first place, they co-operate with the medical man in hospital work. All your readers will know what nursing means by this time. Nursing may be roughly defined as the care of patients under medical control. Such care or treatment is subsidiary simply in the sense that treatment is subsidiary to diagnosis. The doctor in private practice has to learn how to co-ordinate the two. This definition of the status and function of nursing is only another way of laying down a proper division of labour. To give a practical example. Take my own department of the Public Health. I have administrative control over every hospital which the Bristol Public Health Committee has established. In each of these hospitals the resident medical officer has clinical control, while the matron is responsible for the nursing staff. Just as the matron and the nurses may not interfere with the methods of treatment laid down by the medical officer in charge, so the medical officer must leave the nursing arrangements to the matron, except when special circumstances arise calling for his intervention. This principle of the proper division of labour defines the relation of medical men to nurses in hospital or institutional life."

"And what is their relation in general public-health or municipal work?"

"In public-health work I am strongly of opinion that every Health Visitor should be a trained nurse. I am not of opinion that women should be sanitary inspectors. As I said above, those who visit the homes and the mothers should be actual or potential mothers themselves. The lessons of the proper care and feeding of children can best be brought into women's homes by women. These women public Health Visitors should be trained nurses. By the way, the institution of Health Visitors at all is a new departure in Bristol. Looking back over a quarter of a century, for I have been Medical Officer of Health for twenty-six years, I remember that it is eighteen years ago since I first advocated the appointment of Health Visitors. Things have changed very much since then. That advocacy was made in the days of the old Sanitary Committee.

### How a Health Visitor Should be Trained.

Even to-day, however, until quite recently at least, the Midwives Act was worked by the police surgeons. Now, however, two Health Visitors have just been appointed to act under medical supervision. They are the ambassadors of medical science into the homes; furnished too, I may add, with the plenary powers of science if not with the executive powers of Acts of Parliament. Both our Visitors are trained nurses."

"What is the best training for a Health Visitor?"

### Men *versus* Women as Inspectors.

"First, the training of a nurse. That is, in my opinion, an essential. Then, when the three years' training as a nurse is completed, intending Health Visitors should pass the Health Visitors and School Nurses' Examination of the Royal Sanitary Institute. Personally, why women take the Sanitary Institution certificate for inspectors of nuisances I can never understand. For Health Visitors we want trained nurses with a public-health qualification. We do not want practical plumbers. I am of opinion that just as for Health Visitors women are better than men, men are better than women as sanitary inspectors. The ideal Health Visitor is a trained nurse who has gained a Health-Visitor's certificate."

"This covers, then, the relation of medical men to nurses in their second aspect as engaged in municipal work. The present Health Week, as combined with the Conference and Exhibition which has been organised by the Nurses' Social Union, provides a capital example of the immense public gain which results from the relation of doctors and nurses in close co-operation within the limits that I have already laid down."

